# Macrophage Polarization in Chronic Inflammatory Diseases: Killers or Builders?

**DOI:** 10.1155/2018/8917804

**Published:** 2018-01-14

**Authors:** Luca Parisi, Elisabetta Gini, Denisa Baci, Marco Tremolati, Matteo Fanuli, Barbara Bassani, Giampietro Farronato, Antonino Bruno, Lorenzo Mortara

**Affiliations:** ^1^Department of Biomedical, Surgical and Dental Sciences, University of Milan, Milan, Italy; ^2^Immunology and General Pathology Laboratory, Department of Biotechnology and Life Sciences, University of Insubria, Varese, Italy; ^3^Scientific and Technologic Park, IRCCS MultiMedica, Milan, Italy

## Abstract

Macrophages are key cellular components of the innate immunity, acting as the main player in the first-line defence against the pathogens and modulating homeostatic and inflammatory responses. Plasticity is a major feature of macrophages resulting in extreme heterogeneity both in normal and in pathological conditions. Macrophages are not homogenous, and they are generally categorized into two broad but distinct subsets as either classically activated (M1) or alternatively activated (M2). However, macrophages represent a continuum of highly plastic effector cells, resembling a spectrum of diverse phenotype states. Induction of specific macrophage functions is closely related to the surrounding environment that acts as a relevant orchestrator of macrophage functions. This phenomenon, termed polarization, results from cell/cell, cell/molecule interaction, governing macrophage functionality within the hosting tissues. Here, we summarized relevant cellular and molecular mechanisms driving macrophage polarization in “distant” pathological conditions, such as cancer, type 2 diabetes, atherosclerosis, and periodontitis that share macrophage-driven inflammation as a key feature, playing their dual role as killers (M1-like) and/or builders (M2-like). We also dissect the physio/pathological consequences related to macrophage polarization within selected chronic inflammatory diseases, placing polarized macrophages as a relevant hallmark, putative biomarkers, and possible target for prevention/therapy.

## 1. Introduction

Macrophages belong to the mononuclear phagocyte system (MPS), a family of professional phagocytes that includes monocyte and dendritic cells (DCs). Over the past few decades, classification of the cells within the MPS system has generated considerable controversy given the different, often confusing, nomenclature to identify macrophages in different physio/pathological conditions as a consequence of their plasticity, resulting in very different phenotype/functions.

The first open debate arises already in the definition of macrophage cell of origin. The classic scenario of the MPS stated that monocytes recruited from the periphery, under the influence of specific tissue-local growth factors, developed into macrophages. According to this scenario, macrophages derive from hematopoietic progenitors of bone marrow that differentiate under the influence of specific growth factors within the hosting tissues [1]. These cells primarily enter the blood as monocytes and further infiltrate tissues as macrophages, where they adapt to the local microenvironment to play out specific functions, such Kupffer cells in the liver, microglial cells in the brain [2], and mesangial cells in the kidney [3].

This view has been completely reconsidered over the last decade, and the ontogeny of macrophages has been totally rewritten, based on genetic approaches of cell fate mapping. New evidence demonstrated that macrophages can originate from embryonic precursor cells that colonized developing tissues before birth (foetal tissue macrophages) and that tissue-resident macrophages have self-maintaining abilities in the adulthood. Murine models allow the definition of three main sources for tissue-resident macrophages: (i) the yolk sac in the embryo as a source for progenitor cells by primitive hematopoiesis; (ii) the foetal liver, where the hematopoiesis takes places, shifting form the yolk sac, and (iii) the bone marrow that becomes the elicit hematopoietic centre in late embryos and adult organisms [4–6]. Another intriguing scenario, concerning the origin and persistence of macrophages, has been proposed by Gomez et al. [7]. The model proposed that resident macrophages, developing in the embryo independently of the hematopoietic stem cell (HSC) compartment [2, 8–11], still persist in adults and can coexist with the so termed “passenger” leucocytes that include monocytes and DCs, which originated from bone marrow HSCs and myeloid progenitors [1, 12, 13].

The abundance of macrophages within tissues is finely controlled through the axis colony-stimulating factor-1 or macrophage-colony-stimulating factor (CSF-1 or M-CSF), IL-34, and colony-stimulating factor-1 receptor (CSF-1R) [14].

It has been reported that recruited macrophages differ from the resident tissues in terms of transcriptional profiling. Even if the term “macrophage activation” has been commonly used to describe macrophage activity in response to diverse stimuli, several studies pointed out that the results of cell activation deeply depend on the macrophage location and on the stimulus that triggers their activation.

*In vitro* and *in vivo* studies have shown that the phenotypic heterogeneity of macrophages correlates with peculiar functions specific to their local microenvironment [15] and this plasticity enables the appropriate response to pathogen or injury challenge.

Macrophage activation can be obtained in response to a plethora of diverse stimuli, including microbial products, damaged cells, activated lymphocytes, and inflammatory cells, and can result in the acquisition of distinct functional subsets undergoing different phenotypic polarizations.

Macrophage plasticity and heterogeneity give rise to a still opened debate, concerning the nomenclature to identify cell subsets/subtypes undergoing in such different phenotypic, functional (cytokine release), metabolic, regulatory (versus other arms of innate and adaptive immunity) rearrangements.

**Figure 1:**
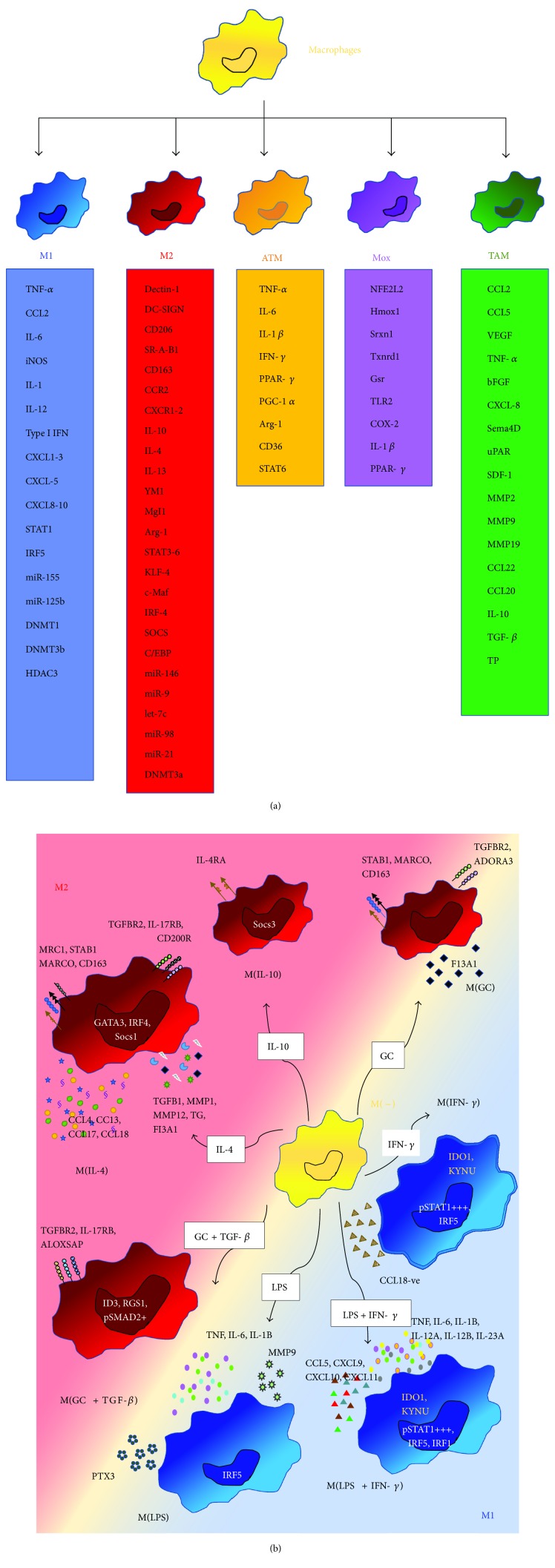
Past and new concept in macrophage polarization. (a) Schematic overview of the different stimuli that can induce the diverse macrophage polarization state. M1: classically activated phenotype; M2: alternatively activated macrophages; ATM: adipose tissue-derived macrophages; Mox: atherosclerosis-associated macrophages; TAMs: tumour-associated macrophages. (b) The polarization landscape of macrophages. According to the different stimulation conditions, macrophages can acquire peculiar M1 or M2 phenotype, governed by the different surface antigen expressions, including scavenger receptors, chemokine, matrix-associated protein and cytokine release, and different patterns of transcription factors and metabolic pathway activated. The driver stimuli include IL-4, IL-10, glucocorticoids (GC) with TGF-*β*, glucocorticoids alone, LPS, LPS and IFN-*γ*, and IFN-*γ* alone.

On the basis of the type-1/type-2 helper- T(h-) cell polarization concept [16, 17], phenotypically polarized macrophages have been defined according to two primary activation states, termed classically activated M1 and alternatively activated M2 (Figure 1(a)). M1 and M2 nomenclature has been long and lastly employed to define the “supposed” main subsets of macrophages, which originates in 2000 by Mills et al. [18]. Basically, M1 and M2 responses exemplify the opposing activities of killing (proinflammatory, “killer M1”) and repairing (anti-inflammatory, “builder M2”) [19].

However, macrophage polarization in many physiologic and pathologic conditions represents a continuum, involving high plasticity and heterogeneity of these effector cells, and resemble mainly to a spectrum of distinct polarization states that do not fit to the oversimplified M1/M2 classification. Hence, in line with a consensus recommendation, we decide to use “M1” to indicate only IFN-*γ* and LPS-driven macrophage phenotypes and “M2” to refer to macrophage phenotypes triggered only by IL 4 or IL 13. Furthermore, we use “M1-like” to illustrate diverse signal-induced polarization states that leads to cell cytotoxic function (killer) and antitumour activities and “M2-like” in relation to distinct phenotypes that share the functional capacity of repair, inducing new vessels and remodelling (builder) in parallel with tumour promotion and immunosuppressive ability toward T-cell responses [20] (Figure 1(b)).

In a normal tissue, the ratio of M1-like/M2-like macrophages is highly regulated and increases during the inflammation process [21]. Gene expression profile analysis showed that M1 macrophages can release high levels of proinflammatory cytokines, including tumour necrosis factor-*α* (TNF-*α*), CCL2 also known as monocyte chemoattractant protein-1 (MCP-1), IL-6, inducible nitric oxide synthase (iNOS), IL-1, IL-12, type I IFNs, CXCL1–3, CXCL5, and CXCL8–10 [22]. On the contrary, M2 macrophages have been demonstrated to express high levels of dectin-1, DC-SIGN (CD209), mannose receptor (CD206), scavenger receptor A, scavenger receptor B-1, CD163, CCR2, CXCR1, and CXCR2 [23] and to produce a large amount of IL-10, YM1, macrophage and granulocyte inducer-form 1 (MgI1), and arginase-1, highlighting their relevance during tissue remodelling and repair [24].

Macrophage polarization and functions are tightly regulated through the activation of several interconnected pathways. Among all, the balance between activation of STAT1 and STAT3/STAT6 has been demonstrated to play a crucial role; indeed, the predominance of STAT1 activation promotes M1 macrophage polarization, resulting in cytotoxic and proinflammatory functions. In contrast, STAT3 and STAT6 activation by IL-4/IL-13 and IL-10 signaling increases M2 macrophage polarization, associated with active tolerance and tissue repairing [22]. Moreover, the downstream effector of STAT6 and KLF-4 promotes M2 macrophage functions by suppressing the NF-*κ*B/HIF-1*α*-dependent transcription. IL-10 promotes M2 polarization inducing p50 NF-*κ*B homodimer, c-Maf, and STAT3 activities. In addition, IL-4 induces c-Myc that activates the IRF4 axis that inhibits IRF5-mediated M1 polarization, resulting in the M2 promotion [22]. Bouhlel et al. also demonstrated the relevance of PPAR-*γ* (peroxisome proliferator-activated receptor gamma) in skewing human monocytes toward an anti-inflammatory M2 phenotype. Indeed, the authors showed that PPAR-*γ* is highly upregulated in M2 macrophages and PPAR-*γ* agonists have been demonstrated to induce directly M2-like differentiation of monocytes *in vivo* and *in vitro* [25].

In the past decade, a novel class of small noncoding RNAs, termed microRNAs (miRs), has emerged as important regulators in biological processes. Accumulating evidence suggest a relevant role for several miRs in the polarization process (Figure 1(a)). In particular, miR-155 and miR-223 are involved in modulating macrophage activation state by targeting SOCS1, C/EBP (a hallmark of M2 macrophages), and Pknox1 [26]. Overexpression or silencing of miR-155 has been demonstrated to drive macrophages to M1 or M2 phenotype, respectively, confirming that miR-155 plays a central role in regulating Akt-dependent M1/M2 polarization of macrophages. It has been also shown that miR-155 downregulates the expression of IL-13R*α*1, suppressing the polarization toward M2 phenotype [27, 28]. Some studies have observed that let-7c was expressed at a higher level in M2 macrophages than in M1 macrophages. Accordingly, the upregulation of let-7c in macrophages diminished M1 phenotype and promotes M2 polarization targeting C/EBP-d [29, 30]. miR-146, miR-125b, miR-155, and miR-9 can inhibit TLR4/IL-1R signaling by regulating IRAK-1, TRAF6, IKKe, p50 NF-*κ*B, and TNF-*α* [29]. Further, miR-17, miR-20a, and miR-106a reduce the expression level of the signal regulatory protein (SIRPa), an important macrophage differentiation-related marker. miR-98 and miR-21 downregulate the expression of inflammatory genes in monocytes and macrophages via controlling IL-10 level [31].

Emerging data have demonstrated that epigenetic mechanisms, including chromatin remodelling, DNA methylation (DNAm), histone modifications, and regulation of target gene expression, are also involved in the orchestration of macrophage polarization in response to local environmental signals [22, 32, 33]. M1 and M2 macrophages have been shown to express different levels of DNA methyltransferase (DNMT) 1, 3a, and b that are associated with gene silencing [34]. DNMT1 drives the M1 polarization in atherosclerosis by directly targeting the promoter of PPAR-*γ* in macrophages [35]. The DNMT3b binding of the promoter of PPAR-*γ* contributes to the M1 phenotype in adipose tissue during inflammatory process [33].

Lund et al. demonstrated that atherogenic lipoproteins can promote global DNA hypermethylation in monocyte [36]. Thus, DNMT inhibition or knockdown could decrease the M1 polarization, providing novel strategies for atherosclerosis prevention and therapy. Accordingly, the treatment with 5-aza-2-deoxycytidine (decitabine), a recognized inhibitor of DNMTs, results in an increased M2 polarization induced by the inhibition of the PPAR-*γ* promoter, which in turn prevents obesity-induced inflammation, atherosclerosis, and insulin resistance [37, 38]. DNMT3a and DNMT3al expression levels have been shown to be increased significantly in M2 compared to M1 macrophages, and this is associated with AMPK signaling [33]. On the contrary, DNMT3b was significantly lower in M2 compared with M1 adipose macrophages [39]. Histone H3 and H4 acetylations were found to be toughly associated with the maturation of human monocytes [40]. M1 polarization induced by IFN-*γ* increases histone H4 acetylation at the TNF-*α* promoter throughout the ERK and p38 mitogen-activated protein kinase (MAPK) signaling pathways [41]. STAT3 and MAPK activation and the simultaneous acetylation of histones H3 and H4 on the SOCS-3 promoter suppress the inflammatory responses in microglial cells and promote M2 polarization [42]. Histone deacetylase 3- (HDAC3-) deficient macrophages showed a decreased expression of IFN-*β* and Cox-1 showing an M2-like phenotype and thereby ameliorate many inflammatory diseases, such as pulmonary inflammation [43–45].

Such heterogeneity in macrophage phenotypes and functions generated the still open questions of whether they act as killers or builders. During inflammation, macrophages drive in the autoregulatory loop characterizing this process, as they release a wide range of biologically active molecules which participated in both detrimental (killers) and beneficial (builders) in inflammation [46–48]. Therefore, inflammation stands as the typical environmental setting where macrophages show their “Janus” behaviour [46–48]. During the first events occurring during inflammation, macrophages are endowed to kill/remove pathogens and damaged cells, while at the end of the inflammatory process, termed resolution of inflammation, macrophages act as builders that promote damaged tissue regeneration and return to homeostasis [49–51]. Since inflammation represents a shared hallmark from diverse chronic diseases and direct involvement in insurgence and progression of these conditions, here, we discuss whether macrophages can act as killers or builders within the inflammatory landscape of selected and apparently “distant” pathologic conditions.

## 2. Macrophages in Cancer: Killers or Builders?

**Figure 2:**
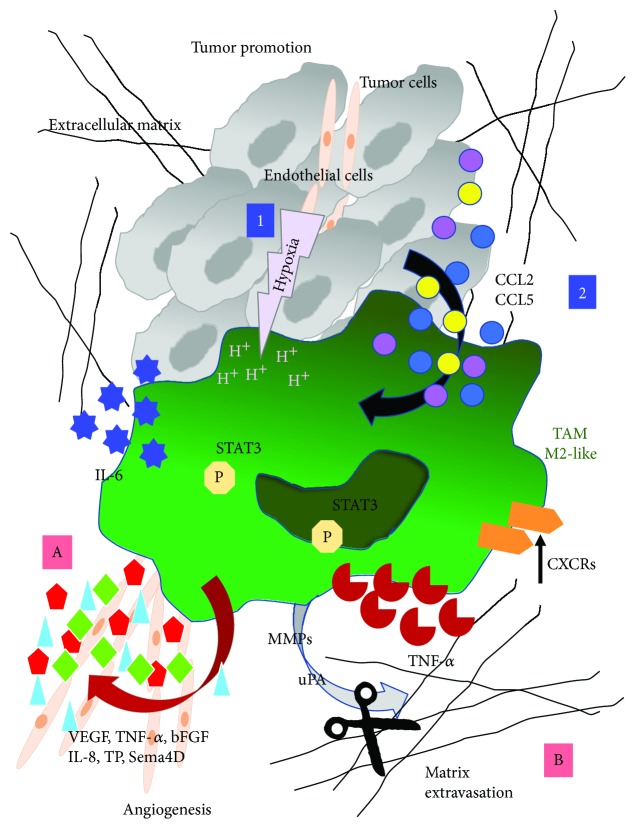
Macrophage polarization in tumour progression. Macrophage recruitment in tumours and their polarization are regulated by several factors. Among all, hypoxia can induce the differentiation of monocytic myeloid-derived suppressor cells (M-MDSCs) via upregulation of CD45 tyrosine phosphatase activity (1). Further, soluble factors, such as CCL2 and CCL5 that are produced by the cancer cells and stroma cells, can increase macrophage infiltrate (2). In the TME, infiltrating associated to tumours (TAM/M2-like macrophages) can orchestrate tumour progression by several mechanisms including the release of cytokine, chemokines, and tissue remodelling proteins. Hypoxia increases the expression of CXCRs in TAMs and promotes tumour angiogenesis by enhancing the production of VEGF, TNF-*α*, bFGF, IL-8, TP, and Sema4D that can induce endothelial cell proliferation, sprouting and migration, tube formation, and maturation of new vessel, followed by its stabilization by attaching mural cells (A). TAMs can regulate the extracellular matrix degradation by producing different types of enzymes and proteases, such as matrix metalloproteinases (MMPs), in particular MMP2, MMP9, plasmin, urokinase plasminogen activator (uPA) and cathepsins acting on connective tissue surrounding the tumour, and allow tumour cells to detach from the mass of origin and to disseminate, leading to the formation of distant metastases (B).

Macrophages represent the most abundant tumour infiltrating inflammatory cells [52, 53]. Reflecting their extreme plasticity within healthy tissues, macrophages infiltrating tumours can acquire distinct phenotype and functions resulting in the attenuation of antitumour activity and induction of tumour-supporting functions and have been defined as tumour-associated macrophages (TAMs) with M2-like features (Figure 2). However, in the initial phases of carcinogenesis, macrophages can act as protective killer cells, cooperating with T lymphocytes in the control of early proliferating cancer cells in the immunoediting process [54]. Instead, in developing tumours, compelling evidence indicate that subverted macrophages or TAMs exert a major role in driving tumour progression by different mechanisms and pathways, depending on the types of tumour, tissues, and inflammatory mediators. The builder option of macrophages in the tumour microenvironment (TME) can lie to conditions in which a chronic nonresolving inflammation is established, a feature that has been defined a hallmark of cancer [55] and that points out TAMs as key inflammatory mediators able to link chronic inflammation with cancer development and progression [56, 57].

Among soluble factors that mediate their displacement, there are CCL2, CCL5, CSF-1, VEGF, and complement elements, which are often produced by the cancer cells and stromal cells in the TME. Moreover, some TAMs can derive from differentiation of monocytic myeloid-derived suppressor cells (M-MDSCs) via upregulation of CD45 tyrosine phosphatase activity in response to tumour hypoxia and following downregulation of STAT3 [58].

Tumour promoting or builder activities exerted by TAMs have been demonstrated by several studies. Elevated TAM infiltration has been correlated with worse clinical outcome in most malignant tumours, such as breast, cervical, ovarian, prostate, and thyroid cancers; Hodgkin's lymphoma; hepatocellular carcinoma; lung carcinoma; and cutaneous melanoma [56, 59–65]. In contrast to these findings, some reports have instead highlighted that tumour infiltrating macrophages correlated to increased survival in colorectal, prostatic, and lung cancer patients [66–70]. The main builder features of TAM include the ability to support tumour angiogenesis as well as lymphangiogenesis, to increase the breakdown of extracellular matrix, to promote tumour cell invasion and migration, and to suppress the antitumour immune responses [56, 62, 71, 72]. These functions are shared with M2-like macrophages that, in a physiological context, are induced during vascular and matrix remodelling, necessary for damage resolution [73–77].

TAM infiltrate is also associated with the onset of resistance to different chemotherapeutic agents through the activation of diverse pathways. In breast cancers, TAMs can induce IL-10/STAT3/Bcl-2 signaling, leading to an inhibition of apoptosis upon paclitaxel treatment [78]. In advanced lung adenocarcinomas, TAMs are also reported to decrease the responsiveness to target therapy based on the epidermal growth factor receptor tyrosine kinase inhibitors [79].

M2-like TAMs support tumour growth directly by producing cytokines able to stimulate the proliferation of tumour cells or indirectly, by fostering endothelial cell (EC) proliferation and angiogenesis (Figure 2). It has been reported that the growth of subcutaneous Lewis lung tumour is impaired in the CSF-1-deficient and macrophage-deficient mice [80]. Furthermore, the treatment of tumour-bearing mice with recombinant CSF-1 reestablished the tumour growth, indicating a role for macrophages in tumour growth. TAMs can produce IL-6, whose release impacts on cell proliferation by a STAT3-dependent mechanism. Inhibition of STAT3 signaling blocks the antiapoptotic activity of IL-6 in human liver cancer cells [81]. TAMs are lower producers of TNF-*α*, resulting in enhanced tumour growth. Hypoxia significantly impacts on the TAM tumour cell interaction that induces the expression of CXCR4 and its ligand, CXCL12 (SDF-1), further supporting tumour cell dissemination and angiogenesis [82]. The number of TAMs within a tumour has been positively correlated with its metastatic potential, suggesting a role for TAMs in the distant dispersion of tumour cells [52, 83, 84]. By producing different types of enzymes and proteases, such as matrix metalloproteinases (MMPs), in particular MMP2 and MMP9, plasmin, urokinase plasminogen activator (uPA), and cathepsins [85–87] (Figure 2), TAMs can regulate the degradation of the extracellular matrix (ECM) and dictate tumour invasion and the metastatic process [19]. These factors act by relaxing the connective tissue surrounding the tumour, allowing tumour cells to detach from the mass of origin and to disseminate, leading to the formation of distant metastases.

TAMs sustain tumour angiogenesis by producing VEGFA (VEGF), the master growth factor involved in the angiogenic process. Besides VEGF, TAMs release a panel of proangiogenic factors which include TNF-*α*, basic fibroblast growth factor (bFGF), CXCL8/IL-8, thymidine phosphorylase (TP), adrenomedullin (ADM), and semaphorin 4D (Sema4D) [88–91] (Figure 2). These factors released by TAMs act by inducing endothelial cell proliferation, sprouting and migration of ECs into the tumour, tube formation, and maturation of new vessel, followed by its stabilization by attaching mural cells [92].

It has been recently reported that the expression of Sema3A from tumour cells is able to promote TAM accumulation inside the tumour, particularly in the avascular areas and required neuropilin-1 (NRP-1)-signaling cascade [93]. Macrophages are not only critical regulators of angiogenesis, but also crucial participants in lymphangiogenesis via VEGFC and VEGFD release, both in inflammatory settings and in tumour progression [94]. Thus, TAM-derived factors can link tumour angiogenesis and lymphangiogenesis [95–97].

Among TAMs, a relevant proangiogenic monocyte/macrophage subset, characterized by some distinctive features, has been further identified. These macrophages can express the angiopoietin receptor Tie2, termed TEMs (Tie2-expressing macrophages), and are closely associated with the vasculature [98, 99]. These cells have been implicated in the interference and in the resistance of action of antiangiogenic therapeutics, in particular vascular disrupting agents, and experimental data support the notion that inhibition of TEMs can foster antiangiogenic treatments with higher inhibition of angiogenesis and tumour spreading [100, 101].

Apart from their extreme plasticity, TAMs also sustain an immunosuppressive milieu aiding tumours to escape from immune surveillance [102]. TAM contribution to tumour progression acts also through synergistic interaction with other arms of the innate and adaptive immunity [46–48, 103] within the immunosuppressive TME. TAMs can interact with MDSCs, neutrophils, and DCs [104, 105]. TAMs also orchestrate the recruitment of T regulatory cells, by secreting CCL20 [106, 107] and CCL22 [108], and their activation through a bidirectional interaction by the release of IL-10 and TGF-*β* [107, 109–111].

Moreover, TAMs represent an important factor for the establishment of the premetastatic niche [112–116].

Different therapeutic strategies have been developed to target TAM physiology with encouraging preclinical and clinical results, either by blocking their tumour recruitment and functions or by redirecting their features to antitumour effector activities [57, 81, 117–121]. In several preclinical experimental models, including prostate, breast, and lung cancer and melanoma, the specific inhibition by antibodies of CCL2 has proven its promising effects, and when they are delivered in combination with chemotherapy shown enhancement of the effectiveness of treatment [122, 123]. However, though in a mouse model of breast cancer, it has been reported that a rebound effect following inhibition of CCL2 pathway resulted in the recruitment of monocytes/macrophages into the tumour and enhancement of lung metastasis [124]; different antibodies targeting CCL2 have been entered phase I and II clinical trials. Regarding the CCL5-CCR5 axis blocking strategies, a CCR5 antagonist has been approved as a treatment for patients with liver metastases of advanced refractory colorectal cancers and preliminary results indicated that this approach can lead to clinical responses [125]. Another interesting TAM-specific therapeutic treatment involves interferences with the CSF-1-CSF-1R axis, and in particular the receptor tyrosine kinase CSF-1R. Several compound and antibody inhibitors have been developed and evaluated in preclinical models and in patients with different types of cancer [120]. Important clinical regressions were obtained from patients with diffuse-type tenosynovial giant-cell tumour, which experienced CSF-1R tumour overexpression [120]. Interestingly, in a mouse glioblastoma multiforme model, CSF-1R blockade did not affect the TAM numbers but instead the M2-like TAM polarization, which is associated with the block of glioma progression and improvement of survival [119]. Also, bisphosphonates, usually used to treat osteoporosis and to prevent bone metastases-related complications, can be used to target macrophages in the tumour context, although their cytotoxic effects have been illustrated initially toward osteoclasts [126]. Combination chemotherapy or hormonal therapy with bisphosphonates in different types of tumour has shown clinical synergistic effects, in particular in postmenopausal women with breast cancer [127]. Another encouraging therapeutic strategy is related to agonistic anti-CD40 antibody and gemcitabine in pancreatic ductal adenocarcinoma patients. This approach revealed clinical responses and importantly demonstrated that in treated mice the CD40 agonist approach is responsible for reeducation of M2-like TAM toward an M1-like phenotype and of effective antitumour responses [128, 129]. Finally, a recently identified compound that found application in soft tissue sarcomas and ovarian cancer patients is trabectedin, which induces selective TRAIL-dependent apoptosis of monocytes, macrophages, and M-MDSCs in the blood, spleens, and tumours with reduction of TAM numbers and angiogenesis [130, 131].

## 3. Macrophages in Type 2 Diabetes: Killers or Builders?

Type 2 diabetes (T2D) is a metabolic disorder, and its incidence has increased significantly in recent years. T2D is characterized by a peripheral resistance to the action of insulin and a failure of beta cells to compensate, leading to hyperglycaemia. It is now widely accepted that obesity increases the risk of T2D by inducing a chronic low-grade inflammation [132] and progression in local adipose tissue.

Accumulating evidence supports a role for tissue macrophages in a broad spectrum of inflammatory conditions [133], including obesity-associated metabolic diseases, such as insulin resistance and T2D [68, 134].

Macrophages together with other immune cells account almost 10% of the normal adipose tissue and play a key role in maintaining homeostasis. However, diet-induced obesity compromises homeostasis, resulting in an increased infiltration of macrophages representing up to 50% of the cells in adipose tissue [135, 136].

**Figure 3:**
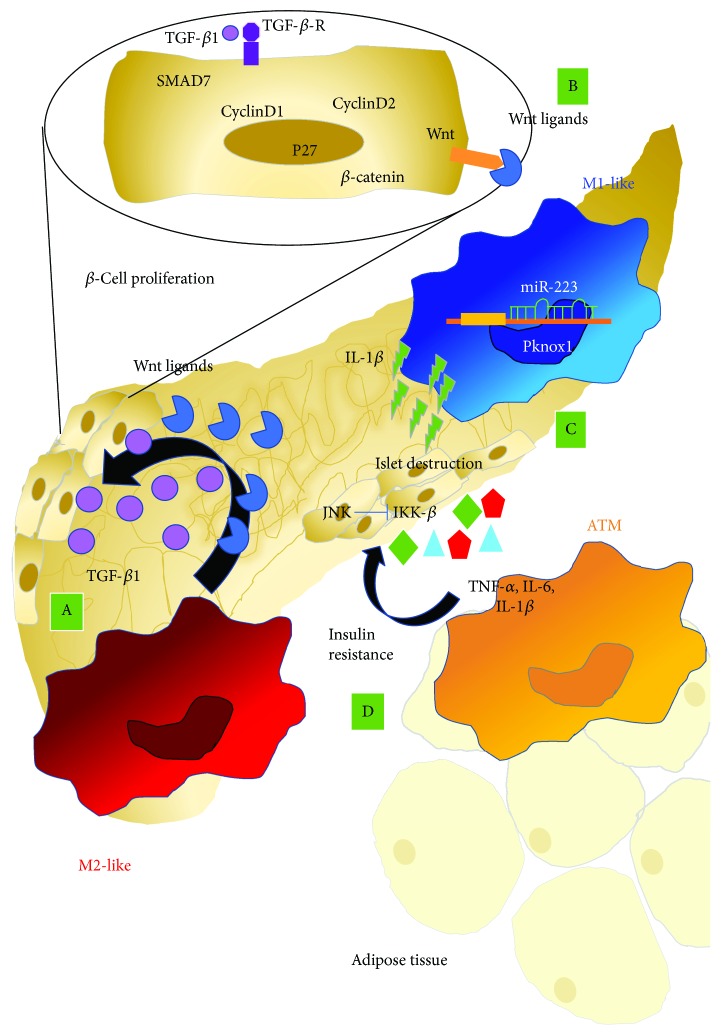
Macrophage polarization in type 2 diabetes. Macrophage within pancreatic tissues can be switched toward different functionalities according to the environment stimuli. M2-like macrophage supports B-cell proliferation by several trophic factors like TGF-*β*1 which directly induce upregulation of SMAD7 and increases of cyclinD1, cyclinD2, and p27 (A). Moreover, M2-like macrophages release Wnt ligands, thus activating the Wnt signaling pathway, and *β*-catenin, supporting *β*-cell replication (B). M1-like macrophage in pancreatic tissues can secrete IL-1b, inhibiting insulin secretion, followed by islet destruction (C). Adipose-derived macrophages (ATM) can release proinflammatory cytokines, including TNF-*α*, IL-6, and IL-1*β* that decrease insulin sensitivity through the activation of Jun N-terminal kinase (JNK), inhibitor of IK*κ*B kinase (IKK-*β*), and other serine kinases in insulin target cells (D).

Several studies have established the crucial role of macrophage polarization in the development of T2D. The M1/M2-like polarization of tissue-destructive (killers) versus tissue-reparative (builders) macrophages is of great interest in clinical strategies because of their role in *β*-cell proliferation [137]. Recent evidence demonstrate that the high plasticity and phenotypic diversity of macrophages promote the cross-talk between *β*-cells, non-*β* endocrine cells, endothelial cells, mesenchymal cells, and other circulation-derived blood cells [138–140]. Builder-M2-like macrophages regulate *β*-cell proliferation through the release of a variety of trophic factors such as TGF-*β*1, which directly induce upregulation of SMAD7 in *β*-cells. SMAD7 in turn promotes *β*-cell proliferation by increasing CyclinD1 and CyclinD2 and by inducing nuclear exclusion of p27 [141] (Figure 3). In addition, M2-like macrophages also secrete Wnt ligands, thus activating the Wnt signaling pathway, and *β*-catenin, supporting *β*-cell replication [138] (Figure 3). Conversely, only a few studies investigating the polarization state of macrophages in pancreatic microenvironment have been described in literature [16–19], where an overall increase of macrophages/islets has been detected by immunohistochemistry. Eguchi et al. [142, 143] showed that Ly6c^+^ M1 macrophage was expanded in the diabetic mouse islet. Ly6c^+^-killer-M1 macrophage has been shown to secrete IL-1*β*, resulting in potent inhibition of insulin secretion, followed by islet destruction (Figure 3). The use of IL-1R antagonists and anti-IL-1*β*-neutralizing antibodies was able to abolish these effects on pancreatic islets [21–24].

Several studies in T2D have shown that M1-like macrophages resulted in increased inflammation, obesity, and insulin resistance, while M2-like macrophages are associated with a reduction in both obesity and insulin resistance [144]. M2-like macrophages are reported to not only suppress inflammatory cytokine IL-10 [145] but also provide a niche for preadipocytes to keep the number and quality of them, thus maintaining insulin sensitivity [146].

These data clearly suggest that macrophages play a nonredundant role in the pathogenesis of T2D [147]. An important aspect of diabetes prevention is a better understanding of the underlying mechanisms behind obesity-induced visceral adipose tissue inflammation, crucial for the development of T2D.

Obesity is associated with the accumulation of proinflammatory cells in visceral adipose tissue, which is an important underlying cause of insulin resistance and progression to T2D [148–150]. Establishing the initiating events leading to the switch from an anti-inflammatory M2-like state to M1-like phenotype remains elusive.

Recent studies show that obesity-induced adipocyte hypertrophy results in upregulated surface expression of stress markers. Adipose stress is detected by local sentinels, such as NK cells and CD8^+^ T cells, which produce IFN-*γ*, driving M1-like adipose tissue macrophage (ATM) polarization [148–150]. Adipocyte hypertrophy has been reported to create hypoxic area and activates hypoxia-inducible factor-1, which induces inflammatory cytokines and suppresses preadipocyte-related angiogenesis and causes insulin resistance [151].

Normal adipose tissue macrophages phenotypically resemble the alternatively activated M2-like phenotype, expressing the mannose receptor, the CD206 surface antigen, and releasing Arg-1 and IL-10. In contrast, diet-induced obesity leads to a shift toward an M1 classically activated macrophage, characterized by the F4/80, CD11b, and CD11c expression [152] (Figure 3).

Low-grade inflammation in this setting is mediated by the polarization of recruited and resident macrophages to the M1-like phenotype in tissues, such as liver and adipose tissues [153, 154]. In contrast, M2 macrophage activation appears to protect against obesity-associated inflammation and insulin resistance [155, 156]. Several cytokines and chemokines, such as CCL2, interleukin IL-6 and IL-1*β*, macrophage migration inhibitory factor (MIF), and TNF-*α*, can be released by both adipocytes and macrophages [157, 158]. Macrophages within adipose tissue are recruited from the bone marrow and are characterized by a wide panel of factors that track with the degree of obesity [136, 159, 160]. Indeed, the paracrine as far as the endocrine activity was exerted by the proinflammatory cytokines, including TNF-*α*, IL-6, and IL-1*β* released by ATMs can induce decreased insulin sensitivity through the activation of Jun N-terminal kinase (JNK), inhibitor of IK*κ*B (IKK-*β*), and other serine kinases in insulin target cells [161, 162].

The unbalance in the ratio between M1-like and M2-like adipose macrophages has been considered to be directly related to the development of insulin resistance [21, 149]. Insulin resistance resulted from a transition in macrophage polarization from the M2-like activation state, induced by STAT6 activation and PPAR, to a classic M1-like activation state, further driven by NF-*κ*B, AP1, and other related factors [163–165].

The network of molecular mediators that regulate M2 polarization in response to hypermetabolism is not fully understood, but peroxisome proliferator-activated receptor gamma coactivator 1-alpha (PGC-1*α*) and PPAR-*γ* target genes, such as arginase-1 and CD36, are implicated in this process. PPAR-*γ* has been proven to be essential for macrophage M2 polarization with the function of anti-inflammation and associated with metabolic dysfunction [145, 156, 166]. PPAR-*γ* was found to be a miR-130b target gene in regulating macrophage polarization insulin tolerance via repression of PPAR-*γ* [167]. Several studies have shown that PPAR-*γ* interacts with NF-*κ*B, in the modulation of macrophage polarization. PPAR-*γ* blocked the proinflammatory pathway of NF-*κ*B and inhibited the expression of relative factors, such as TNF-*α* [168].

Further, it was shown that IL-6 acts as a Th2-builder cytokine in obesity by stimulating M2-like polarization and local ATM proliferation, presumably due to upregulation of the IL-4 receptor *α* [169]. Recently, it has been reported that adenosine monophosphate kinase (AMPK) *β*1 plays an important role in protecting macrophages from inflammation under high lipid exposure resulting in a modulation of obesity-induced insulin resistance (Figure 3). Genetic deletion of the AMPK *β*1 subunit in mice reduced macrophage AMPK activity, acetyl-CoA carboxylase phosphorylation, and mitochondrial content, resulting in reduced rates of fatty acid oxidation [170].

Inhibition of proinflammatory cytokines and chemokines, such as TNF-*α*, IL-1*β*, IL-6, and CCL2, may reduce adipose tissue inflammation and insulin resistance [147, 171, 172]. For instance, several studies have demonstrated that treatment with neutralizing IL-1*β* antibody or blockage of IL-1*β* signaling improved glycaemic control in diet-induced obese mice and insulin sensitivity in patients with T2D [173–176]. Other findings suggest that the CCL2-CCR2 signaling pathway disruption reduces adipose tissue macrophage content ameliorating insulin resistance and improves insulin sensitivity [160, 177]. CCL2 knockout mice receiving intact monocytes or mice receiving CCR2-deficient monocytes were both protected from the accumulation of macrophages in adipose tissue and the liver. [178] So far, targeting the CCL2-CCR2 signaling pathway may provide the basis for the development of novel therapies against T2D. *In vivo* studies have shown that circulating levels of free fatty acid (FFA) promote the generation of M1 macrophages via TLR4 signaling in adipocytes and macrophages in the setting of obesity [179–181]. In this context, adipose tissue inflammation is aggravated by the secretion of TNF-*α*, which in turn increases lipolysis leading to further production of FFAs establishing a vicious circle. Resistin is another potential target to combat insulin resistance or T2D. In fact, resistin induction which in turn stimulates secretion of several proinflammatory cytokines by increased infiltration of macrophages causes inflammation-induced insulin resistance [182–184].

Several phase II and III clinical trials have been initiated to inhibit key immunological processes of adipose tissue inflammation in T2D patients, such as NF-*κ*B signaling, IL-1*β* function, or arachidonic acid metabolism, with promising results [148].

A shift in the polarization of adipose tissue macrophages from an M2-like state to an M-like proinflammatory state resulting in insulin resistance favours inflammation and insulin resistance [145]. Thus, targeting of inflammatory M1/M2-like polarization process of obese patients appears to be a promising future strategy for prophylaxis against diabetes development. For instance, adipose tissue macrophages from CCR2 knockout mice are polarized to the M2-like macrophages, even after obesity and CCR2 knockout mice were found to be protected from diet-induced insulin resistance [145, 160]. Furthermore, it has been shown that inhibition of IL-10 secreted by M2-like macrophages enhances the impairment of insulin signaling confirming its protective role in T2D [185].

Insulin-sensitizing thiazolidinediones (TZDs), clinically used for T2D patients [186], target the PPAR-*γ* that plays a key role in the maturation of M2-like macrophage and insulin sensitivity. PPAR-*γ* deletion prevents polarization of the monocyte/macrophage to the M2-like phenotype, and PPAR-*γ*-deficient mice exhibit glucose intolerance and insulin resistance [187]. Therefore, existing and future drug mechanisms may be involved in modulating the phenotypical and functional features of macrophages. For instance, metformin is a drug widely used to treat T2D, to decrease insulin resistance; it has been proposed that the benefit may result, at least in part, from modulating macrophage differentiation and polarization [188, 189]. How metformin can modulate the differentiation of Ly6C monocytes into M2-like macrophages remains the subject of ongoing interesting studies. In addition to glucose-lowering drugs, T2D patients are typically treated with low-dose aspirin (acetylsalicylic acid) that has off-target anti-inflammatory properties. Aspirin exerts its anti-inflammatory effects via inhibition of cyclooxygenase and a subsequent decrease in the proinflammatory prostaglandins [190]. Recently, it has been demonstrated that aspirin-triggered resolvin D1 into a degradable biomaterial after injury was able to significantly increase the accumulation of anti-inflammatory monocytes and M2-like macrophages while limiting the infiltration of neutrophils and increase proregenerative immune subpopulations [191].

Incretin-based treatments and the cannabinoid 1 receptor (CB1) blocker rimonabant have anti-inflammatory effects and may protect the pancreatic islets from IL-1*β*-driven. However, this anorectic antiobesity and glucose-lowering drug had also psychiatric side effects [164, 192, 193].

Several studies highlight the role of miRs as key regulators of cell fate determination and significant contributors to the pathogenesis of complex diseases, such as inflammatory responses and T2D [194]. It was found that miR-223 inhibits Pknox1, suppressing proinflammatory activation of macrophages, and protects against diet-induced adipose tissue inflammatory response and systemic insulin resistance [195]; miR-130b was found to be a novel regulator of macrophage polarization via repression of PPAR-*γ* and a promising target for T2D therapy [167]; miR-27a was also proposed as a target of intervention for inflammation and insulin resistance in obesity [196].

In summary, M1/M2-like macrophage polarization and switching hold the key to the regulation of insulin sensitivity and T2D. Macrophage polarization toward the alternative M2-like phenotype may play a preventive role and also be a novel and useful strategy for the treatment of insulin resistance and T2D.

Novel macrophage-targeted strategies that are both tissue-specific and disease-specific hold a promise for the future management of the chronic inflammatory disorders that were covered in this review.

## 4. Macrophages in Atherosclerosis: Killers or Builders?

**Figure 4:**
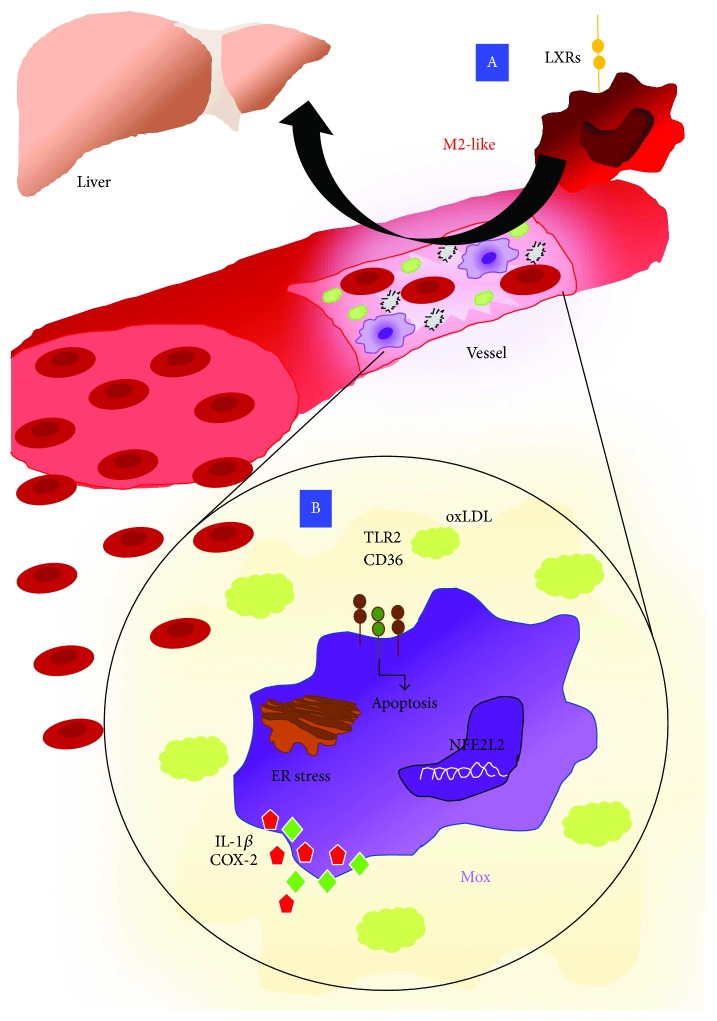
Macrophage polarization in atherosclerosis. Macrophages are crucial players involved in the atherosclerosis development due to their ability to regulate cholesterol efflux. In this context, the upregulation of LXRs in M2 macrophages has been found to exert a protective role. Indeed, LRXs reduce peripheral tissue excess cholesterol that is returned to the liver by releasing HDL in the plasma (A). Apart from M1 and M2 polarization, a third macrophage state has been described in the atherosclerosis context that is termed Mox. Macrophages exposed to oxidized phospholipids display reduced phagocytic and chemotactic abilities compared with M1- and M2-like macrophages and are characterized by the expression of the transcription factor NFE2L2 as far as Hmox1, Srxn1, Txnrd1, and Gsr genes. Mox macrophages also activate TLR2­dependent mechanisms in response to oxidized lipids leading to an increase of IL­1*β* and COX-2 (B).

Atherosclerosis is a chronic inflammatory disease driven by an imbalance in lipid metabolism and a maladaptive immune response [197]. This disease is characterized by the accumulation of lipids in large- and medium-sized arteries forming plaque deposits that block the flow of the blood. Several factors have been correlated with the development of atherosclerotic diseases, among which the elevated low-density lipoprotein (LDL) cholesterol, hypertension, obesity, and both T2D and T1D. The accumulation of LDL promotes the recruitment of monocytes that lead to the formation of the atherosclerotic plaques [198]. Further, the exposure to CSF-1 and the uptake of oxidized LDL (ox-LDL) induce monocyte differentiation into macrophage and results in foam cell formation with the proliferation of smooth muscle cells [199]. The scavenger receptors lead the ox-LDL recognition, and the intracellular cholesterol is metabolized and transported to exogenous acceptors, such as high-density lipoprotein, through efflux proteins, such as ATP-binding cassette transporters [200] (Figure 4).

Macrophage apoptosis has been observed in patients with defects in the Acyl-CoA:cholesterol acyltransferase (ACAT), the enzyme that re-esterificates free cholesterol in cholesteryl fatty acid esters [198]. Seimon et al. showed that oxidized phospholipids, oxidized LDL, saturated fatty acids (SFAs), and lipoprotein(a) can induce apoptosis in ER-stressed macrophages through a CD36- and TLR2-dependent mechanism [201] (Figure 4).

Several *in vivo* studies have demonstrated macrophage heterogeneity within the atherosclerotic plaque in response to the exposition of lipids and their oxidized derivatives [202]. Indeed, within atherosclerotic microenvironment, macrophages adapt their phenotype activating specific transcriptional programs. Cholesterol crystals that accumulate during the early stages of the atherosclerotic process might be involved in the activation of macrophages [202]. Cholesterol crystals can promote the caspase1-activating NLRP3 inflammasome, which results in the cleavage and secretion of IL-1 and may act as a M1-polarizing stimulus [203]. The proinflammatory M1-like phenotype can also be promoted by a mechanism that involves inhibition of the transcription Kruppel-like factor 2 [204, 205] or the activation of the TLR4-mediated pathway that in turn leads to the activation of NF­*κ*B [206]. Conversely, the anti-inflammatory M2-like phenotype is induced by 9-oxononanoyl cholesterol, a major cholesteryl ester oxidation product that can enhance TGF­*β* secretion [207]. Moreover, sphingolipid metabolites, such sphingosine­1­phosphate (S1P), promote the switching phenotype of mouse macrophages from M1- to M2-like state, by activating S1P1 receptor [208].

Recently, a third macrophage phenotype has been described in the atherosclerosis context that has been termed Mox (Figure 4) and represents macrophages exposed to oxidized phospholipids [209–211]. In advanced atherosclerotic lesions of mice, Mox macrophages comprise approximately 30% of the total number of macrophages [212]. Mox phenotype can be triggered by the activation of transcription factor NFE2L2 [212, 213]. Mox macrophages display reduced phagocytic and chemotactic abilities compared with M1- and M2-like macrophages. In mice, Mox macrophages typically express NFE2L2-mediated redox regulatory genes, including *Hmox1*, *Srxn1*, *Txnrd1*, and *Gsr* [212]. Nevertheless, in response to oxidized phospholipids, Mox macrophages activate TLR2-dependent mechanisms that lead to an increase of IL-1*β* and COX-2 expression [214].

Circulating monocytes in murine models have been classified into two major subsets, described as Ly6C^hi^ and Ly6C^low^ monocytes. In apolipoprotein E-deficient (ApoE^−/−^), mice the increase of Ly6C^hi^ subset (corresponding to human M1-like subset) has been observed within atherosclerotic plaques [215].

Several studies have also correlated macrophage polarization with the clinical course of atherosclerosis. Among all, de Gaetano et al. [216] observed a marked difference in a macrophage subset between symptomatic and asymptomatic plaques. Indeed, M1 macrophages were found to be abundant in the developed lipid core of the symptomatic plaque and were rarely found in the intimal regions of the plaque, while M2-like macrophage number was higher in asymptomatic atherosclerotic plaques, suggesting a potential protective role of M2-like macrophages. Moreover, in mouse models, it has been demonstrated that in the regressing plaque a decrease in the number of macrophages occurs and, in some, a switch of their phenotypic characteristics has been observed, with an enrichment in M2-like phenotype, suggesting that this is a common signature of regressing plaques [217].

Despite several current standard therapies for atherosclerosis that may influence general immune responses, including angiotensin-converting enzyme (ACE) inhibitors, *β*-blockers, aspirin, and corticosteroids, these drugs lack specific macrophage targeting and may only be recognized as mild modifiers of macrophage activity [218]. Several common pharmacological agents have already been proposed to modulate macrophage activity for the prevention as well as the treatment of inflammatory-related diseases, including atherosclerosis. PPAR-*γ* is a crucial factor involved in the regulation of macrophage lipid metabolism and inflammatory responses and, as already discussed above, is upregulated in M2-like macrophages [25]. PPAR-*γ* activators might have therapeutic potential, and studies conducted by Bai et al. [219] suggest that mediator 1 (MED1) is required for the PPAR-*γ*-induced M2 phenotype switch and showed that MED1 in macrophages has an antiatherosclerotic activity via PPAR-*γ*-regulated transactivation, suggesting MED1 as a promising target for atherosclerosis therapy.

Natural ligands such prostaglandins and some pharmacological agents including anti-TZD that have been demonstrated to activate PPAR-*γ* have also been shown to decrease atherosclerosis progression. Choi et al. demonstrated that 5-(4-hydroxy-2,3,5-trimethylbenzylidene) thiazolidine-2,4-dione (HMB-TZD) reduced leukotriene B4 (LTB4) production and cytokine production by RAW264.7 macrophages and attenuates atherosclerosis possibly by reducing monocyte recruitment to the lesion [220]. In *in vivo* studies, selective inactivation of macrophage PPAR-*γ* impairs M2-like activation exacerbating diet-induced obesity [154], suggesting that PPAR-*γ* inducer might have a therapeutic potential. Likewise, liver X receptors (LXRs) have been found to be upregulated in M2-like macrophages and exert atheroprotective effects by modulating cholesterol metabolism and M1 macrophage-induced inflammatory genes, including iNOS, COX-2, and IL-6 [221] (Figure 4). Tangirala et al. have observed that in experimental models of atherosclerosis, LXR agonists induced a reduction of preexisting plaque size and this was associated with LXR macrophage activity. Indeed, macrophage-specific loss of LXRs resulted in a statistically significant increase in lesion size [222]. Moreover, the immunomodulatory drug fingolimod (FTY720) that has been described as a S1P1 receptor modulator has been shown to increase the proportion of M2-like macrophages in atherosclerotic lesions and reduce lesion progression in mice [223]. Statins, effective cholesterol-lowering agents, have also been reported to dampen immune responses through inhibition of macrophage inflammatory activity by increasing efferocytosis *in vitro* in a 3-hydroxyl-3-methylglutaryl coenzyme A (HMG-CoA) reductase-dependent manner, decreasing membrane localization of RhoA and preventing impaired efferocytosis by lysophosphatidic acid, a potent inducer of RhoA [224].

Stimulation of the macrophage autophagy-lysosomal system by the natural sugar trehalose has been reported to reduce the formation of the atherosclerotic plaque by limiting macrophage apoptosis and necrosis in the plaque cores [225].

Finally, some *Lactobacillus* has been observed to regulate M1/M2-like macrophage ratio by suppressing ox-LDL phagocytosis, thus blocking foam cell formation [226]. These data supported the employment of prebiotic or probiotic in atherosclerosis.

## 5. Macrophages in Periodontitis: Killers or Builders?

Gingivitis and periodontitis are two common diseases affecting the oral tissues and the health of the supporting structures of a tooth that share inflammation as a common feature. While in gingivitis the inflammatory process is limited to the soft tissues, epithelium, and connective tissue, in periodontitis, the inflammation is extended to the supporting tissues, including the alveolar bone [227].

Chronic periodontitis (CPD) occurs in response to specific bacteria within the oral biofilm and involves the destruction of tooth-supporting tissues. Major features for CPD are accumulation of immune cells in gingival connective tissue, resorption of alveolar bone, and the degradation of periodontal connective tissues, which lead to increased tooth mobility and eventual tooth loss [228, 229].

Chronic periodontitis is strongly associated with the presence of Gram-negative anaerobic bacteria in subgingival plaque, in particular, *Porphyromonas gingivalis*, *Tannerella forsythia*, and *Treponema denticola*. Although initiated by bacteria, the bone pathology in CPD is mediated almost entirely by the host response that is thought to be responsible for the local tissue destruction observed in periodontitis [230]. In addition, the response to oral pathogens has systemic consequences. For example, infection and chronic inflammatory conditions, such as periodontitis, may influence the atherogenic process [231, 232].

It has been reported that monocyte/macrophages act as relevant killers in periodontal diseases by contributing to tissue breakdown. Elevated numbers of macrophages/monocytes associated with greater collagen breakdown and higher level of MMPs have been observed in samples from periodontitis [233]. Studies have shown that IL-1 was expressed predominantly by macrophages in the tissue isolated from periodontal patients [234]. In addition, higher levels of Receptor activator of nuclear factor kappa-B ligand (RANKL) protein, associated with macrophages, have been observed in the periodontitis tissues [235].

Activated macrophages have been found in the gingival epithelium, *lamina propria*, and perivascular tissues and in the blood vessels in human CPD. As lesions are associated with chronic periodontitis progress, increasing numbers of macrophages infiltrate into the gingival tissues [236]. Therefore, the gingival tissue and crevicular fluid of patients with chronic periodontitis have been reported to contain significantly increased amounts of CCL3, also known as macrophage inflammatory protein- (MIP-) 1*α* and CXCL-8/IL-8, as compared to healthy subjects [237, 238].

**Figure 5:**
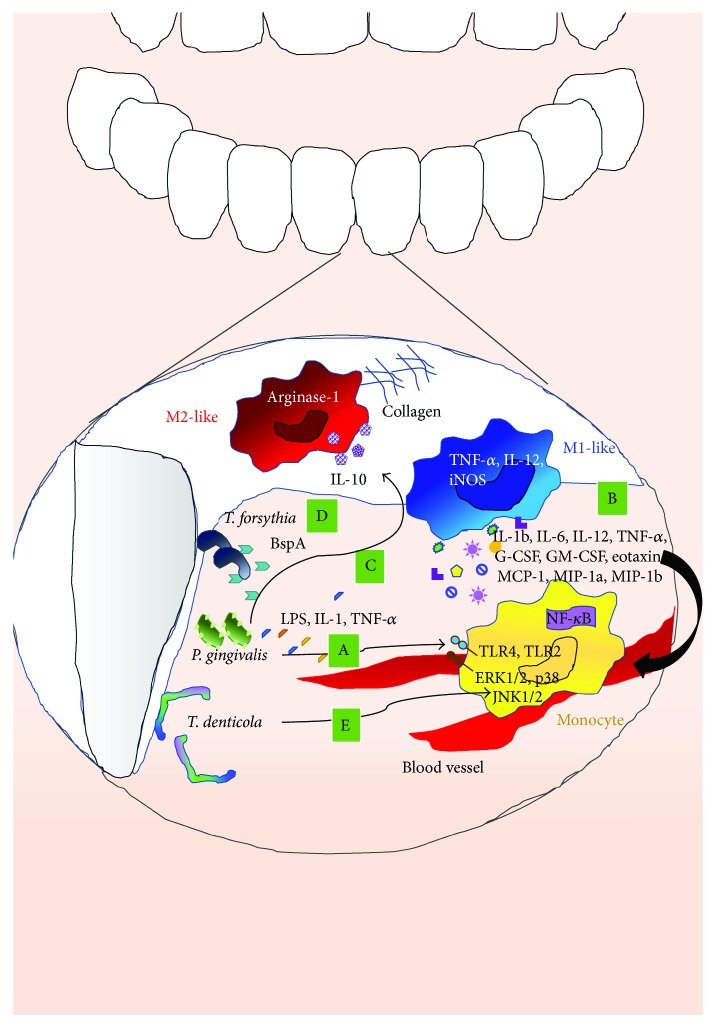
Macrophage polarization in periodontitis. Macrophages that have been found in the gingival epithelium can be activated by several microorganisms able to induce macrophage polarization toward M1- or M2-like phenotype. *P. gingivalis* releases LPS, IL-1, and TNF-*α* that promote the proinflammatory M1 macrophage polarization (A). Moreover, Pg infection enhances the secretion of IL-1*β*, IL-6, IL-12, TNF-*α*, G-CSF, GM-CSF, and the chemokines eotaxin, MCP1, MIP-1*α*, and MIP-1*β* from macrophages, reflecting a M1-like proinflammatory response (B). In spite of this, it has also been reported that *Pg* infection can also be associated with the increase of IL10, supporting M2 macrophage and increasing arginase-1 production and collagen deposition, leading to periodontitis (C). *T. forsythia* releases BspA and other ligands that induce TLR2 signaling favouring the development of Th2-type inflammatory responses (D). *T. denticola* induces TLR2 signaling that stimulates the prolonged activation of both ERK1/2 p38 and JNK1/2 in monocytes (E).

*Porphyromonas gingivalis* (*Pg*) is a key periodontal pathogen that promotes dysbiosis between host-and plaque-associated bacteria, thus resulting in both periodontal disease onset and progression [239, 240]. LPS from *Pg* activates macrophages through both TLR2 and TRL4 [241], and specifically, TLR2 activation by *Pg* LPS triggers the downstream stimulation of NF-*κ*B, leading to the production of proinflammatory cytokines [242–244] (Figure 5).

Macrophages are frequently used as the *in vitro* model cells to define immune cell function in CPD studies. Transfer of TLR2 expressing macrophages to TLR2-deficient mice restored host sensitivity to *Pg* oral challenge [245] (Figure 5).

*Pg* LPS, in the presence of IL-1 and TNF-*α*, has been shown to induce cultured human fibroblasts and epithelial cells to release PGE2, a factor associated with periodontal bone resorption that promotes the proinflammatory M1-like macrophage polarization [229, 246–250] (Figure 5). IL-1 and TNF-*α* not only enhance inflammation but also promote bone resorption, a major concern in periodontitis [251–253]. Oral infection with *Pg* in BALB/c and C57BL/6 mice resulted in the influx of M1 macrophages into the submandibular lymph node (SMLN) and gingival tissue, together with an increase in alveolar bone resorption, as compared with untreated mice in a murine model of periodontitis [254, 255]. Selective SMLN macrophage *in vivo* depletion, using liposomes containing the proapoptotic agent clodronate, resulted in decreased *Pg*-induced alveolar bone *in vivo* resorption.

*Pg* infection enhances the secretion of the cytokines IL-1*β*, IL-6, IL-12, TNF-*α*, CSF-3 (G-CSF), and CSF-2 (GM-CSF), in addition to the chemokines eotaxin and CCL2–4 from macrophages, reflecting a M1 proinflammatory response (Figure 5). These cytokines and chemokines are known to act as proinflammatory mediators, to induce monocytes to migrate from the bloodstream into the gingival tissue, and to act synergistically to further stimulate proinflammatory cytokine production [246, 248, 249, 256]. IL-10, which is mainly produced by macrophages, was detected among the wide array of cytokines released during *Pg* infection [257]. IL-10 strongly supports M2-like macrophage and polarized functions including increased production of arginase-1, higher collagen deposition, and induction of fibrosis in gingival tissue, all common clinical features of chronic periodontitis [258–260].

In a recent study, Lam et al. observed that *Pg* can persist in naïve and M2-like, but not M1-like, macrophages for 24 hours. Phagocytosis of *Pg* also induced high levels of TNF-*α*, IL-12, and iNOS in M1 macrophages, but not in naïve macrophages (MØ) or M2 macrophages [254].

*T. forsythia* expresses a well-characterized TLR2 ligand, the BspA protein, and N- and O-glycan-linked glycoproteins that comprise its surface- (S-) layer, covering the outer membrane [261]. This S-layer has been shown to be important in delaying the cytokine responses of monocyte and macrophage cells *in vitro* [262, 263]. BspA and other ligands of *T. forsythia* induce TLR2 signaling favoring the development of Th2-type inflammatory responses detrimental to the alveolar bone that has been shown to be limited in TLR2^−/−^ mice [242].

*T. forsythia* whole cells induced significantly greater amounts of IL-6 and IL-10 in wild-type (BALB/c) bone marrow-derived dendritic cells (BM-DCs) and macrophages, markers related to an M2-like polarization, as compared with TLR2^−/−^ cells. The macrophage-inducible C-type lectin receptor (Mincle), a Fc*γ*R-coupled pathogen recognition receptor (PRR) [263, 264], has been reported to contribute to macrophage polarization [265]. THP-1 macrophages infected with the purified S-layer on whole wild-type *T. forsythia* elicit a M2-like polarization (IL-10, TNF-*α*) that is limited in Mincle knockdown macrophages or where infection is performed with the S-layer TfΔtfsAB-mutated form [265] (Figure 5).

*Treponema denticola* is among the most frequently isolated oral spirochetal species in patients with periodontitis [266, 267]. Major surface protein complex (MSPc), which is expressed on the envelope of this treponema, plays a key role in the interaction between *T. denticola* and gingival cells and the related cytopathic effects [268]. *Treponema denticola* within the periodontium of the host has been reported to be associated with localized inflammation. MSPc has been showed to stimulate the release of the proinflammatory cytokines NO, TNF-*α*, and IL-1*β* from murine macrophages, both in LPS-responsive and LPS-nonresponsive murine macrophages [269]. Furthermore, IL-1*β*, IL-6, and TNF-*α* secretion by *T. denticola*-activated macrophages has been shown to exhibit potent bone reabsorption effects due to their proosteoclastic properties [270].

*T. denticola-*mediated macrophage response is mainly mediated by TLR2 and via MAP kinases [271]. One of the most highly conserved signaling cascades activated in both the innate and the adaptive immune systems involves a family of MAPKs including ERK1/2, p38, and JNK1/2 [272].

*T. denticola* stimulates the prolonged activation of both ERK1/2 and p38 in monocytes, and pharmacological inhibition of these pathways plays major roles in regulating both pro- and anti-inflammatory cytokine productions by *T. denticola*-stimulated monocytes [271] (Figure 5).

A study from Miyajima et al. reported a correlation between periodontitis-activated monocytes/macrophages and aortic inflammation in an *in vivo* ligature-induced experimental model of periodontitis. Gene expression profiling in circulating monocytes in this experimental model showed that periodontitis induced a M1-like specific signature with high levels of TNF-*α* and IL-6 as compared to controls, indicating that a M1-like phenotype of macrophages is induced by periodontitis [273]. This in turn supports the hypothesis that periodontitis-induced M1-like macrophages are the inflammatory orchestrators driving specific proinflammatory messages to the systemic vasculature [273]. The work from Miyajima et al. also showed that periodontitis-induced M1 macrophages can increase macrophage adhesion to aortic endothelial cells through the NF-*κ*B/VCAM-1 axis [273]. These results clearly suggest that local-tissue alterations of macrophages during periodontitis can impact on circulating monocyte polarization and are associated to vascular alterations involved in apparently distant pathologies that shares inflammatory cell polarization as common features.

## 6. Conclusion

It is now widely accepted that inflammation represents a host hallmark of diverse chronic diseases, ranging from cancer, diabetes, and metabolic, cardiovascular, and neurological/neurodegenerative disorders. In the same way, inflammation has been recognized as a relevant condition for insurgence, maintenance, and progression of such disorders. Cell plasticity is a key and shared feature of inflammatory cells within the host organism that can potentially acquire killer (M1-like) or builder (M2-like) properties, based on the surrounding environment. Macrophages are the clearest example of immune cells that can be switched from killers to builders and vice versa, and this has been observed in all the inflammatory-based/associated disorders. Here we discussed the cellular and molecular mechanisms involved in macrophage switching to killers or builders in differently and apparently distant disorders, pointing out the attention on how the macrophages/microenvironment reciprocal interaction shape their polarization and distinct functional states.

Further, we discussed some approaches aimed at resolving this process, by interfering with aberrant macrophage killer/builder reciprocal switch. With this knowledge, it is clear that the identification of novel preventive and intervention strategies, along with effective compounds able in targeting/limiting/reverting proinflammatory macrophage polarization, are urgently needed and may represent a relevant tool to shape macrophage function action directly on them or on the hosting/surrounding environment.

## Abbreviations

ACAT: Acyl-CoA:cholesterol acyltransferase
ADM: Adrenomedullin
AKT: Protein kinase B
AMPK: Adenosine monophosphate kinase
ATMs: Adipose tissue macrophages
Bcl2: B-cell lymphoma 2
bFGF: Basic fibroblast growth factor
BspA: Bark storage protein A
c-Maf: Avian musculoaponeurotic fibrosarcoma oncogene homolog
c-Myc: Avian myelocytomatosis viral oncogene homolog
C/EBP: CCAAT-enhancer-binding proteins
CCR: Chemokine receptor
CD: Cluster of differentiation
COX: Cyclooxygenase
CPD: Chronic periodontitis
CSF-1: Colony-stimulating factor 1
CSF-1R: Colony-stimulating factor 1 receptor
CXCL: C-X-C chemokine ligand
CXCR: C-X-C chemokine receptor
DC-SIGN: Dendritic cell-specific ICAM-grabbing nonintegrin
DC: Dendritic cells
DNAm: DNA methylation
DNMT: DNA methyltransferase
ECM: Extracellular matrix
ER: Endoplasmic reticulum
ERK: Extracellular signal-regulated kinase
G-CSF: Granulocyte colony-stimulating factor
GM-CSF: Granulocyte macrophage colony-stimulating factor
GSR: Glutathione-disulfide reductase
HDAC: Histone deacetylase
HIF: Hypoxia-inducible factor
HMB-TZD: 5-(4-Hydroxy-2,3,5-trimethylbenzylidene) thiazolidine-2,4-dione
HMG-CoA: 3-Hydroxyl-3-methylglutaryl coenzyme A
Hmox1: Heme oxygenase 1
IFN: Interferon
IKK: I*κ*B kinase
IKK*β*: Inhibitor of IK*κ*B kinase
IL: Interleukin
iNOS: Inducible nitric oxide synthase
IRAK: Interleukin receptor-associated kinase
IRF: Interferon regulatory factor
JNK: Jun N-terminal kinase
KLF4: Kruppel-like factor 4
LDL: Low-density lipoprotein
let-7: Lethal-7
LPS: Lipopolysaccharide
LTB4: Leukotriene B4
LXRs: Liver X receptors
M-CSF: Macrophage colony-stimulating factor
M: Macrophage
MAPK: Mitogen-activated protein kinase
MCP: Monocyte chemoattractant protein
MCP1: Monocyte chemoattractant protein-1
MDSCs: Myeloid-derived suppressor cells
MED1: Mediator 1
MgI1: Macrophage and granulocyte inducer-form 1
MIF: Macrophage migration inhibitory factor
Mincle: Macrophage-inducible C-type lectin receptor
MIP: Macrophage inflammatory protein
miRNA/miR: Micro-RNA
MMPs: Metalloproteases
MØ: Naïve macrophages
Mox: Macrophages exposed to oxidized phospholipids
MPS: Mononuclear phagocyte system
MSPc: Major surface protein complex
NF-*κ*B: Nuclear factor kappa-light-chain-enhancer of activated B-cells
NFE2L2: Nuclear factor- (erythroid-derived 2) like 2
NLRP3: NLR family pyrin domain containing 3
NO: Nitric oxide
NRP1: Neuropilin-1
ox-LDL: Oxidized LDL
PD: Periodontitis
*Pg*: *Porphyromonas gingivalis*
PGC-1*α*: Peroxisome proliferator-activated receptor gamma coactivator 1-alpha
PGE2: Prostaglandin E2
Pknox1: PBX/knotted 1 homeobox 1
PPAR: Peroxisome proliferator-activated receptor
PRR: Pathogen recognition receptor
RANTES: Regulated on activation, normal T cell expressed and secreted
S1P: Sphingosine­1­phosphate
SDF-1: Stromal cell-derived factor-1
Sema4D: Semaphorin 4D
SFAs: Saturated fatty acids
SIRPa: Signal-regulatory protein
SMAD: Small mother against decapentaplegic
SMLN: Submandibular lymph node
SOCS1: Suppressor of cytokine signaling 1
Srxn1: Sulfiredoxin-1
STAT: Signal transducers and activators of transcription
T2D: Type 2 diabetes
TAM: Tumour-associated macrophage
TEMs: Tie2-expressing monocytes
TGF: Transforming growth factor
Th: T helper
TLR: Toll-like receptor
TNF: Tumour necrosis factor
TP: Thymidine phosphorylase
TRAF: TNF receptor-associated factor
Txnrd1: Thioredoxin reductase 1
TZD: Thiazolidinediones
uPA: Urokinase-type plasminogen activator
VCAM: Vascular cell adhesion molecule
VEGF: Vascular endothelial growth factor
YM1: Chitinase 3-like 3.
